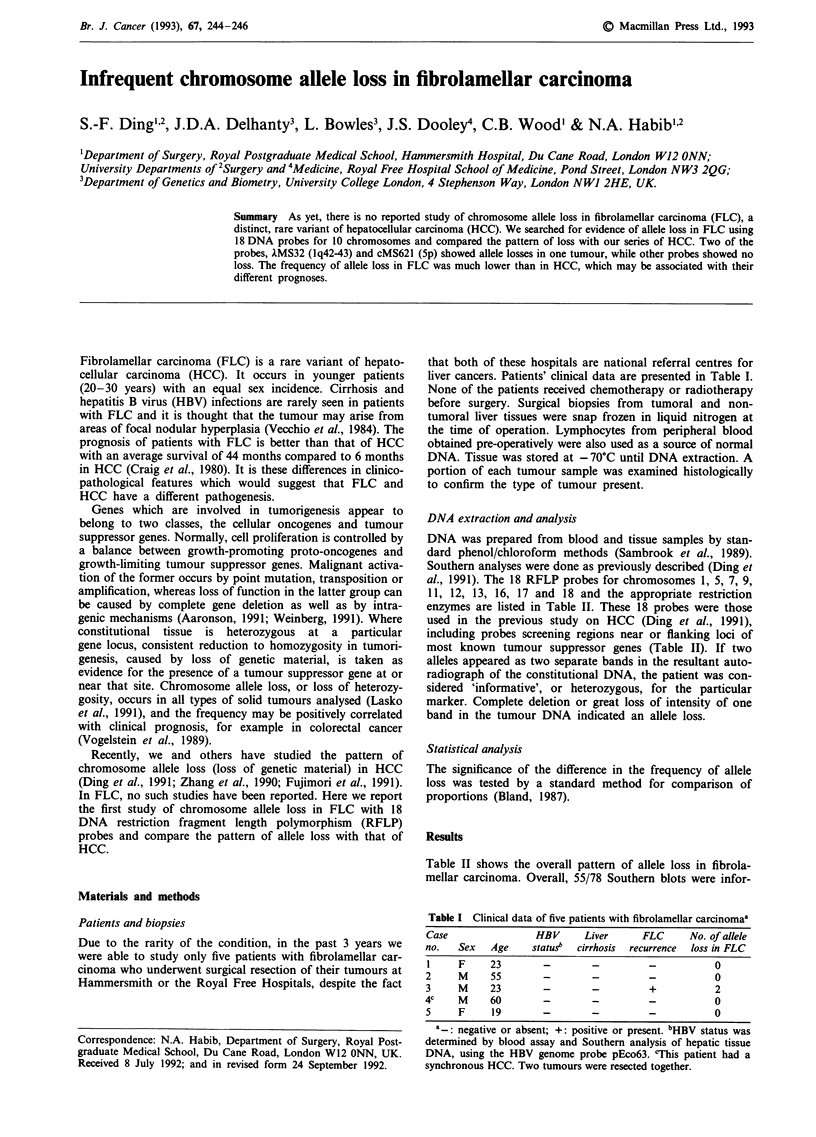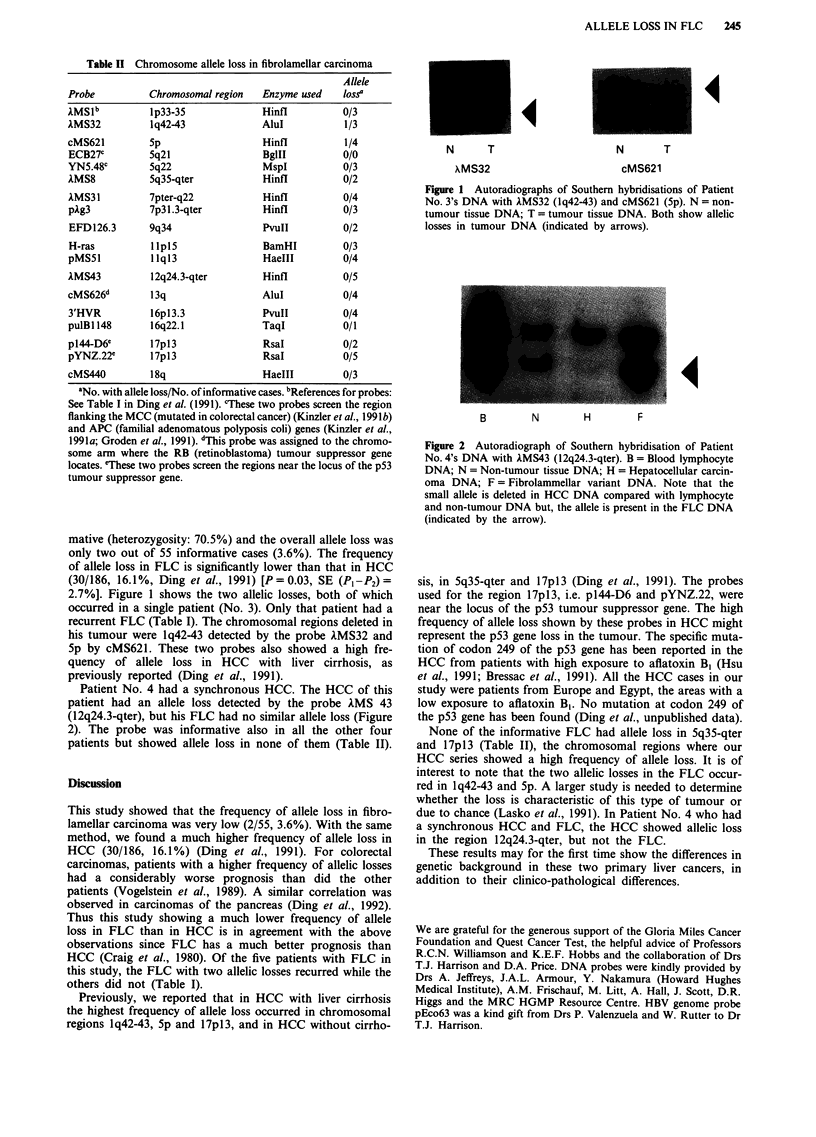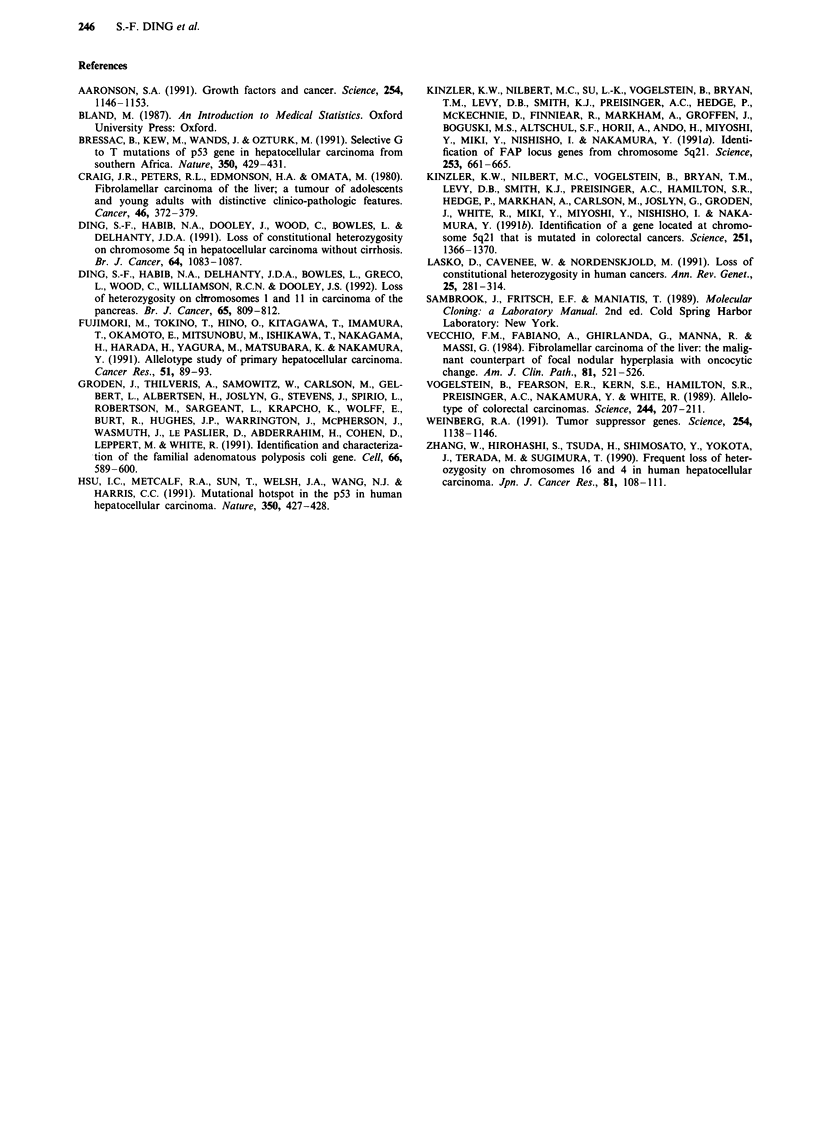# Infrequent chromosome allele loss in fibrolamellar carcinoma.

**DOI:** 10.1038/bjc.1993.47

**Published:** 1993-02

**Authors:** S. F. Ding, J. D. Delhanty, L. Bowles, J. S. Dooley, C. B. Wood, N. A. Habib

**Affiliations:** Department of Surgery, Royal Postgraduate Medical School, Hammersmith Hospital, London, UK.

## Abstract

**Images:**


Br. J. Cancer (1993), 67, 244-246 © Macmillan Press Ltd., 1993

### Infrequent chromosome allele loss in fibrolamellar carcinoma

S.-F. Ding[1,2], J.D.A. Delhanty[3], L. Bowles[3], J.S. Dooley[4], C.B. Wood[1] & N.A. Habib[1,2]

[1]*Department of Surgery, Royal Postgraduate Medical School, Hammersmith Hospital, Du Cane Road, London W12 0NN; University Departments of* [2]*Surgery and* [4]*Medicine, Royal Free Hospital School of Medicine, Pond Street, London NW3 2QG;* [3]*Department of Genetics and Biometry, University College London, 4 Stephenson Way, London NW1 2HE, UK.*

**Summary** As yet, there is no reported study of chromosome allele loss in fibrolamellar carcinoma (FLC), a distinct, rare variant of hepatocellular carcinoma (HCC). We searched for evidence of allele loss in FLC using 18 DNA probes for 10 chromosomes and compared the pattern of loss with our series of HCC. Two of the probes, λMS32 (1q42-43) and cMS621 (5p) showed allele losses in one tumour, while other probes showed no loss. The frequency of allele loss in FLC was much lower than in HCC, which may be associated with their different prognoses.

Fibrolamellar carcinoma (FLC) is a rare variant of hepatocellular carcinoma (HCC). It occurs in younger patients (20–30 years) with an equal sex incidence. Cirrhosis and hepatitis B virus (HBV) infections are rarely seen in patients with FLC and it is thought that the tumour may arise from areas of focal nodular hyperplasia (Vecchio *et al.*, 1984). The prognosis of patients with FLC is better than that of HCC with an average survival of 44 months compared to 6 months in HCC (Craig *et al.*, 1980). It is these differences in clinicopathological features which would suggest that FLC and HCC have a different pathogenesis.

Genes which are involved in tumorigenesis appear to belong to two classes, the cellular oncogenes and tumour suppressor genes. Normally, cell proliferation is controlled by a balance between growth-promoting proto-oncogenes and growth-limiting tumour suppressor genes. Malignant activation of the former occurs by point mutation, transposition or amplification, whereas loss of function in the latter group can be caused by complete gene deletion as well as by intragenic mechanisms (Aaronson, 1991; Weinberg, 1991). Where constitutional tissue is heterozygous at a particular gene locus, consistent reduction to homozygosity in tumorigenesis, caused by loss of genetic material, is taken as evidence for the presence of a tumour suppressor gene at or near that site. Chromosome allele loss, or loss of heterozygosity, occurs in all types of solid tumours analysed (Lasko *et al.*, 1991), and the frequency may be positively correlated with clinical prognosis, for example in colorectal cancer (Vogelstein *et al.*, 1989).

Recently, we and others have studied the pattern of chromosome allele loss (loss of genetic material) in HCC (Ding *et al.*, 1991; Zhang *et al.*, 1990; Fujimori *et al.*, 1991). In FLC, no such studies have been reported. Here we report the first study of chromosome allele loss in FLC with 18 DNA restriction fragment length polymorphism (RFLP) probes and compare the pattern of allele loss with that of HCC.

### Materials and methods

### *Patients and biopsies*

Due to the rarity of the condition, in the past 3 years we were able to study only five patients with fibrolamellar carcinoma who underwent surgical resection of their tumours at Hammersmith or the Royal Free Hospitals, despite the fact that both of these hospitals are national referral centres for liver cancers. Patients' clinical data are presented in Table I. None of the patients received chemotherapy or radiotherapy before surgery. Surgical biopsies from tumoral and nontumoral liver tissues were snap frozen in liquid nitrogen at the time of operation. Lymphocytes from peripheral blood obtained pre-operatively were also used as a source of normal DNA. Tissue was stored at −70°C until DNA extraction. A portion of each tumour sample was examined histologically to confirm the type of tumour present.

### *DNA extraction and analysis*

DNA was prepared from blood and tissue samples by standard phenol/chloroform methods (Sambrook *et al.*, 1989). Southern analyses were done as previously described (Ding *et al.*, 1991). The 18 RFLP probes for chromosomes 1, 5, 7, 9, 11, 12, 13, 16, 17 and 18 and the appropriate restriction enzymes are listed in Table II. These 18 probes were those used in the previous study on HCC (Ding *et al.*, 1991), including probes screening regions near or flanking loci of most known tumour suppressor genes (Table II). If two alleles appeared as two separate bands in the resultant autoradiograph of the constitutional DNA, the patient was considered 'informative', or heterozygous, for the particular marker. Complete deletion or great loss of intensity of one band in the tumour DNA indicated an allele loss.

### *Statistical analysis*

The significance of the difference in the frequency of allele loss was tested by a standard method for comparison of proportions (Bland, 1987).

### Results

Table II shows the overall pattern of allele loss in fibrolamellar carcinoma. Overall, 55/78 Southern blots were infor-

**Table I** Clinical data of five patients with fibrolamellar carcinoma[a]

| *Case no.* | *Sex* | *Age* | *HBV status*[b] | *Liver cirrhosis* | *FLC recurrence* | *No. of allele loss in FLC* |
|---|---|---|---|---|---|---|
| 1 | F | 23 | − | − | − | 0 |
| 2 | M | 55 | − | − | − | 0 |
| 3 | M | 23 | − | − | + | 2 |
| 4[c] | M | 60 | − | − | − | 0 |
| 5 | F | 19 | − | − | − | 0 |

[a]−: negative or absent; +: positive or present. [b]HBV status was determined by blood assay and Southern analysis of hepatic tissue DNA, using the HBV genome probe pEco63. [c]This patient had a synchronous HCC. Two tumours were resected together.

Correspondence: N.A. Habib, Department of Surgery, Royal Postgraduate Medical School, Du Cane Road, London W12 0NN, UK.
Received 8 July 1992; and in revised form 24 September 1992.

**Table II** Chromosome allele loss in fibrolamellar carcinoma

| *Probe* | *Chromosomal region* | *Enzyme used* | *Allele loss*[a] |
|---|---|---|---|
| λMS1[b] | 1p33-35 | HinfI | 0/3 |
| λMS32 | 1q42-43 | AluI | 1/3 |
| cMS621 | 5p | HinfI | 1/4 |
| ECB27[c] | 5q21 | BglII | 0/0 |
| YN5.48[c] | 5q22 | MspI | 0/3 |
| λMS8 | 5q35-qter | HinfI | 0/2 |
| λMS31 | 7pter-q22 | HinfI | 0/4 |
| pλg3 | 7p31.3-qter | HinfI | 0/3 |
| EFD126.3 | 9q34 | PvuII | 0/2 |
| H-ras | 11p15 | BamHI | 0/3 |
| pMS51 | 11q13 | HaeIII | 0/4 |
| λMS43 | 12q24.3-qter | HinfI | 0/5 |
| cMS626[d] | 13q | AluI | 0/4 |
| 3'HVR | 16p13.3 | PvuII | 0/4 |
| pulB1148 | 16q22.1 | TaqI | 0/1 |
| p144-D6[e] | 17p13 | RsaI | 0/2 |
| pYNZ.22[e] | 17p13 | RsaI | 0/5 |
| cMS440 | 18q | HaeIII | 0/3 |

[a]No. with allele loss/No. of informative cases. [b]References for probes: See Table I in Ding *et al.* (1991). [c]These two probes screen the region flanking the MCC (mutated in colorectal cancer) (Kinzler *et al.*, 1991*b*) and APC (familial adenomatous polyposis coli) genes (Kinzler *et al.*, 1991*a*; Groden *et al.*, 1991). [d]This probe was assigned to the chromosome arm where the RB (retinoblastoma) tumour suppressor gene locates. [e]These two probes screen the regions near the locus of the p53 tumour suppressor gene.

**Figure 1** Autoradiographs of Southern hybridisations of Patient No. 3's DNA with λMS32 (1q42-43) and cMS621 (5p). N = non-tumour tissue DNA; T = tumour tissue DNA. Both show allelic losses in tumour DNA (indicated by arrows).

**Figure 2** Autoradiograph of Southern hybridisation of Patient No. 4's DNA with λMS43 (12q24.3-qter). B = Blood lymphocyte DNA; N = Non-tumour tissue DNA; H = Hepatocellular carcinoma DNA; F = Fibrolammellar variant DNA. Note that the small allele is deleted in HCC DNA compared with lymphocyte and non-tumour DNA but, the allele is present in the FLC DNA (indicated by the arrow).

mative (heterozygosity: 70.5%) and the overall allele loss was only two out of 55 informative cases (3.6%). The frequency of allele loss in FLC is significantly lower than that in HCC (30/186, 16.1%, Ding *et al.*, 1991) [$P = 0.03$, SE $(P_1 - P_2) = 2.7\%$]. Figure 1 shows the two allelic losses, both of which occurred in a single patient (No. 3). Only that patient had a recurrent FLC (Table I). The chromosomal regions deleted in his tumour were 1q42-43 detected by the probe λMS32 and 5p by cMS621. These two probes also showed a high frequency of allele loss in HCC with liver cirrhosis, as previously reported (Ding *et al.*, 1991).

Patient No. 4 had a synchronous HCC. The HCC of this patient had an allele loss detected by the probe λMS 43 (12q24.3-qter), but his FLC had no similar allele loss (Figure 2). The probe was informative also in all the other four patients but showed allele loss in none of them (Table II).

### Discussion

This study showed that the frequency of allele loss in fibrolamellar carcinoma was very low (2/55, 3.6%). With the same method, we found a much higher frequency of allele loss in HCC (30/186, 16.1%) (Ding *et al.*, 1991). For colorectal carcinomas, patients with a higher frequency of allelic losses had a considerably worse prognosis than did the other patients (Vogelstein *et al.*, 1989). A similar correlation was observed in carcinomas of the pancreas (Ding *et al.*, 1992). Thus this study showing a much lower frequency of allele loss in FLC than in HCC is in agreement with the above observations since FLC has a much better prognosis than HCC (Craig *et al.*, 1980). Of the five patients with FLC in this study, the FLC with two allelic losses recurred while the others did not (Table I).

Previously, we reported that in HCC with liver cirrhosis the highest frequency of allele loss occurred in chromosomal regions 1q42-43, 5p and 17p13, and in HCC without cirrhosis, in 5q35-qter and 17p13 (Ding *et al.*, 1991). The probes used for the region 17p13, i.e. p144-D6 and pYNZ.22, were near the locus of the p53 tumour suppressor gene. The high frequency of allele loss shown by these probes in HCC might represent the p53 gene loss in the tumour. The specific mutation of codon 249 of the p53 gene has been reported in the HCC from patients with high exposure to aflatoxin $B_1$ (Hsu *et al.*, 1991; Bressac *et al.*, 1991). All the HCC cases in our study were patients from Europe and Egypt, the areas with a low exposure to aflatoxin $B_1$. No mutation at codon 249 of the p53 gene has been found (Ding *et al.*, unpublished data).

None of the informative FLC had allele loss in 5q35-qter and 17p13 (Table II), the chromosomal regions where our HCC series showed a high frequency of allele loss. It is of interest to note that the two allelic losses in the FLC occurred in 1q42-43 and 5p. A larger study is needed to determine whether the loss is characteristic of this type of tumour or due to chance (Lasko *et al.*, 1991). In Patient No. 4 who had a synchronous HCC and FLC, the HCC showed allelic loss in the region 12q24.3-qter, but not the FLC.

These results may for the first time show the differences in genetic background in these two primary liver cancers, in addition to their clinico-pathological differences.

We are grateful for the generous support of the Gloria Miles Cancer Foundation and Quest Cancer Test, the helpful advice of Professors R.C.N. Williamson and K.E.F. Hobbs and the collaboration of Drs T.J. Harrison and D.A. Price. DNA probes were kindly provided by Drs A. Jeffreys, J.A.L. Armour, Y. Nakamura (Howard Hughes Medical Institute), A.M. Frischauf, M. Litt, A. Hall, J. Scott, D.R. Higgs and the MRC HGMP Resource Centre. HBV genome probe pEco63 was a kind gift from Drs P. Valenzuela and W. Rutter to Dr T.J. Harrison.

### References

AARONSON, S.A. (1991). Growth factors and cancer. *Science*, **254**, 1146–1153.

BLAND, M. (1987). *An Introduction to Medical Statistics*. Oxford University Press: Oxford.

BRESSAC, B., KEW, M., WANDS, J. & OZTURK, M. (1991). Selective G to T mutations of p53 gene in hepatocellular carcinoma from southern Africa. *Nature*, **350**, 429–431.

CRAIG, J.R., PETERS, R.L., EDMONSON, H.A. & OMATA, M. (1980). Fibrolamellar carcinoma of the liver; a tumour of adolescents and young adults with distinctive clinico-pathologic features. *Cancer*, **46**, 372–379.

DING, S.-F., HABIB, N.A., DOOLEY, J., WOOD, C., BOWLES, L. & DELHANTY, J.D.A. (1991). Loss of constitutional heterozygosity on chromosome 5q in hepatocellular carcinoma without cirrhosis. *Br. J. Cancer*, **64**, 1083–1087.

DING, S.-F., HABIB, N.A., DELHANTY, J.D.A., BOWLES, L., GRECO, L., WOOD, C., WILLIAMSON, R.C.N. & DOOLEY, J.S. (1992). Loss of heterozygosity on chromosomes 1 and 11 in carcinoma of the pancreas. *Br. J. Cancer*, **65**, 809–812.

FUJIMORI, M., TOKINO, T., HINO, O., KITAGAWA, T., IMAMURA, T., OKAMOTO, E., MITSUNOBU, M., ISHIKAWA, T., NAKAGAMA, H., HARADA, H., YAGURA, M., MATSUBARA, K. & NAKAMURA, Y. (1991). Allelotype study of primary hepatocellular carcinoma. *Cancer Res.*, **51**, 89–93.

GRODEN, J., THILVERIS, A., SAMOWITZ, W., CARLSON, M., GELBERT, L., ALBERTSEN, H., JOSLYN, G., STEVENS, J., SPIRIO, L., ROBERTSON, M., SARGEANT, L., KRAPCHO, K., WOLFF, E., BURT, R., HUGHES, J.P., WARRINGTON, J., MCPHERSON, J., WASMUTH, J., LE PASLIER, D., ABDERRAHIM, H., COHEN, D., LEPPERT, M. & WHITE, R. (1991). Identification and characterization of the familial adenomatous polyposis coli gene. *Cell*, **66**, 589–600.

HSU, I.C., METCALF, R.A., SUN, T., WELSH, J.A., WANG, N.J. & HARRIS, C.C. (1991). Mutational hotspot in the p53 in human hepatocellular carcinoma. *Nature*, **350**, 427–428.

KINZLER, K.W., NILBERT, M.C., SU, L.-K., VOGELSTEIN, B., BRYAN, T.M., LEVY, D.B., SMITH, K.J., PREISINGER, A.C., HEDGE, P., MCKECHNIE, D., FINNIEAR, R., MARKHAM, A., GROFFEN, J., BOGUSKI, M.S., ALTSCHUL, S.F., HORII, A., ANDO, H., MIYOSHI, Y., MIKI, Y., NISHISHO, I. & NAKAMURA, Y. (1991*a*). Identification of FAP locus genes from chromosome 5q21. *Science*, **253**, 661–665.

KINZLER, K.W., NILBERT, M.C., VOGELSTEIN, B., BRYAN, T.M., LEVY, D.B., SMITH, K.J., PREISINGER, A.C., HAMILTON, S.R., HEDGE, P., MARKHAN, A., CARLSON, M., JOSLYN, G., GRODEN, J., WHITE, R., MIKI, Y., MIYOSHI, Y., NISHISHO, I. & NAKAMURA, Y. (1991*b*). Identification of a gene located at chromosome 5q21 that is mutated in colorectal cancers. *Science*, **251**, 1366–1370.

LASKO, D., CAVENEE, W. & NORDENSKJOLD, M. (1991). Loss of constitutional heterozygosity in human cancers. *Ann. Rev. Genet.*, **25**, 281–314.

SAMBROOK, J., FRITSCH, E.F. & MANIATIS, T. (1989). *Molecular Cloning: a Laboratory Manual*. 2nd ed. Cold Spring Harbor Laboratory: New York.

VECCHIO, F.M., FABIANO, A., GHIRLANDA, G., MANNA, R. & MASSI, G. (1984). Fibrolamellar carcinoma of the liver: the malignant counterpart of focal nodular hyperplasia with oncocytic change. *Am. J. Clin. Path.*, **81**, 521–526.

VOGELSTEIN, B., FEARSON, E.R., KERN, S.E., HAMILTON, S.R., PREISINGER, A.C., NAKAMURA, Y. & WHITE, R. (1989). Allelotype of colorectal carcinomas. *Science*, **244**, 207–211.

WEINBERG, R.A. (1991). Tumor suppressor genes. *Science*, **254**, 1138–1146.

ZHANG, W., HIROHASHI, S., TSUDA, H., SHIMOSATO, Y., YOKOTA, J., TERADA, M. & SUGIMURA, T. (1990). Frequent loss of heterozygosity on chromosomes 16 and 4 in human hepatocellular carcinoma. *Jpn. J. Cancer Res.*, **81**, 108–111.